# Understanding adolescent health risk behaviour and socioeconomic position: A grounded theory study of UK young adults

**DOI:** 10.1111/1467-9566.13240

**Published:** 2021-02-26

**Authors:** Laura Tinner, Deborah Caldwell, Matthew Hickman, Rona Campbell

**Affiliations:** ^1^ Population Health Sciences Bristol Medical School University of Bristol Bristol UK

**Keywords:** adolescence, ALSPAC, health behaviour, inequality, qualitative, social class, socioeconomic status, young adult, young people

## Abstract

Health risk behaviours such as tobacco smoking, excessive alcohol consumption, drug use, unhealthy diet and unprotected sexual intercourse contribute to the global burden of non‐communicable diseases and are often initiated in adolescence. An individualistic focus on ‘health risk behaviours’ has resulted in behaviour change strategies that are potentially ineffective and increase inequalities. We conducted a grounded theory study of 25 young adults to increase the limited qualitative evidence base surrounding young people, health risk behaviours and socioeconomic inequalities. We found that health risk behaviours were perceived as class markers, manifesting as class stigma, leading some participants from lower socioeconomic backgrounds to employ strategies to avoid such behaviours. Peers and family were core constructs for understanding the relationship between health risk behaviours and socioeconomic life trajectories. However, individualism and choice were consistently expressed as the overriding narrative for understanding health risk behaviour and socioeconomic position during the transition to adulthood. The use of ‘*personal responsibility*’ discourse by young adults, we argue, highlights the need for a public health focus on achieving structural changes as opposed to individualised approaches to avoid reinforcing neoliberal ideologies that serve to marginalise and maintain social inequalities.

## BACKGROUND

Health risk behaviours such as tobacco smoking, excessive alcohol consumption, drug use, unhealthy diet and unprotected sexual intercourse are global health issues, contributing to a range of non‐communicable diseases (James et al., 2018). However, sociological critique highlights how the public health emphasis on ‘individual behaviours’ supports neoliberal ideology, whereby, individuals think of themselves as morally responsible for the prevention of personal illness through avoiding risks and making ‘healthy’ choices (Cohn, 2014; Morris, 2017). This attention on health risk behaviours is reflected in the dominance of individual ‘behaviour change’ interventions, which are increasingly challenged over concerns about ineffectiveness (Frohlich & Abel, 2014) and the potential for increasing inequalities through marginalising those who are unable to change their behaviour (Lupton, 1993). There has been little attention on how health risk behaviour is conceptualised by lay people (Cohn, 2014), which may provide insight into the limitations of public health strategies.

We acknowledge the conceptual shift towards ‘health practices’ over ‘health behaviours’ within social science (Cohn, 2014) and that the phrase ‘health risk behaviours’ is more common within epidemiology. Although we used the more neutral term ‘health behaviours’ in interviews to avoid judgement of participants, we adopt the term ‘health risk behaviours’ in this paper to reflect our specific interest with behaviours that are harmful to health, not just behaviours broadly related to health.

The social circumstances within which we ‘grow, live, work and age’ or the ‘social determinants of health’ are widely understood to be responsible for the social gradient in health (Marmot et al., 2010). There is considerable evidence and literature surrounding health risk behaviours and inequality (Currie et al., 2008; Huijts et al., 2017; Pickett et al., 2005), largely stemming from epidemiological attempts to describe how social determinants, such as socioeconomic position (SEP), impact on inequality in health outcomes, with less attention being paid to the processes leading to these health inequalities (Frohlich & Abel, 2014). While quantitative mapping is hugely important (e.g. Wilkinson and Pickett (2008), Marmot and Shipley (1996)), it occurs alongside theoretical debates surrounding the ‘erosion of class identities’ in late modernity, in which some have questioned the usefulness of SEP as an objective or meaningful social identifier (Pakulski & Waters, 1996). Nevertheless, the consistency of the social gradient in health over time can be taken as evidence that inequalities are not an artefact of epidemiological methods (Macintyre, 1997). The fact there has been little improvement but rather widening inequalities in the UK highlights the need to explore ways to address them.

Health risk behaviours are often initiated in adolescence (Sawyer et al., 2012) (defined here as approximately aged 12–19 years), meaning that this age group is increasingly the target of public health intervention. Such interventions are largely based on the idea of teaching adolescents that they can avoid certain behaviours and make positive choices, instilling the notion that individuals are personally responsible for a safe and successful transition into adulthood (Renedo et al., 2020). This understanding of health risk behaviours as individual ‘lifestyle choices’ ignores the environmental, cultural and socioeconomic factors shaping health, downplays the context within which these behaviours are performed (Campbell, 2001) and further reinforces neoliberal ideology. However, there is little empirical evidence on whether these interventions actually increase inequalities (Tinner et al., 2018). There have been strategies that recognise the web of inter‐connected factors that link adolescent health risk behaviours and deprivation. The Teenage Pregnancy Strategy, for example, was implemented in England to reduce teenage pregnancy and support young parents (Hadley et al., 2016). It suggested that multi‐component, structural interventions that address socioeconomic factors may have greater success in improving adolescent health than individualised educational programmes. This example signifies the potential benefit in structural approaches for other health risk behaviours such as anti‐social behaviour and physical inactivity, which we know are also related to socioeconomic deprivation (Hair et al., 2009).

More broadly, adolescents and young adults (defined here as approximately between 18 and 29 years old) have been underrepresented in inequalities research, which is surprising given the opportunities for intergenerational social mobility through education and entry to the labour market (Karvonen et al., 1999). Qualitative work has also been sparse, with researchers having called for young people to have a stronger voice, seeing this as key to improving health (Sawyer et al., 2012). As such, we enquire whether health risk behaviours initiated during adolescence are intertwined with social inequalities and explore the extent to which they are ‘major contributors to links between deprivation and inequality in later life’ (Viner et al., 2018).

Notwithstanding the issues related to the conceptual focus on ‘health risk behaviours’, these behaviours remain a societal concern. There is strong evidence they predict social problems like unemployment (Kempf‐Leonard et al., 2001) and police arrests (Hair et al., 2009). Health risk behaviours are also associated with major public health issues in the UK such as obesity (Campbell et al., 2020). Thus, there is justification for intervening on adolescent health risk behaviours to improve future health and socioeconomic circumstances of individuals but also reduce the impact on wider society. Further, understanding how people conceptualise health risk behaviours and how they relate to socioeconomic circumstances is critical to informing more equitable policies (Holmes et al., 2017).

We chose the term ‘socioeconomic position’, which has been used extensively in inequalities research to indicate factors such as education, occupational status and income, with debates surrounding this term explored in the literature (Karvonen & Rahkonen, 2011). We also use ‘middle class’ and ‘working class’ as these were more readily adopted by participants. These class identities partially reflect socioeconomic categories of employment, with ‘working classes’ traditionally occupying manual jobs and ‘middle classes’ tending signify the ‘professions’ requiring higher education. However, these classes go beyond static indicators. They are ‘sites for political struggle’ and are discursively adopted to signify (a lack of) ‘taste’, knowledge and the ‘right ways of being and doing’ (Bourdieu, 1984; Lawler, 2005). Further, we recognise the complexities surrounding these ‘classes’, that they are only ‘on paper’ and are relational and mediated through the participants’ conceptualisations (Thirlway, 2020).

To address the gaps in the literature, this qualitative study explored the following research question: How do young adults understand adolescent health risk behaviours in relation to socioeconomic position? To answer this question, we sought to understand the extent to which young adults view (lack of) engagement in health risk behaviours during adolescence as having contributed to their and their peers’ socioeconomic circumstances in young adulthood; specifically, their education and employment. Secondly, we explored whether young adults view their parents’ socioeconomic position as influencing their own adult socioeconomic position and/or their engagement in health risk behaviour.

## METHODS

### Design and sample

The study used semi‐structured interviews of 25 young adults recruited from The Avon Longitudinal Study of Parents and Children (ALSPAC) birth cohort, which has biological and behavioural data from before birth to early adulthood (Fraser et al., 2012). We nested our study in the birth cohort, meaning we could access the cohort participants to theoretically sample based on previously collected data. ALSPAC recruited 14,541 pregnant women residing in and around the City of Bristol, UK with expected dates of delivery 1st April 1991 to 31st December 1992 (Boyd et al., 2013). The use of the ALSPAC cohort reflected our desire to interview young adults as they would be mature enough to reflect on their adolescent health risk behaviours and the impact on their recent transition into adulthood, while still being able to remember the experiences of their youth.

Participants were eligible for the study if they had completed a questionnaire on health risk behaviours during adolescence and we had data on their mother's education (n = 2,204). To maintain confidentiality of the larger cohort study, sampling was handled by the ALSPAC data team so the study team were blind to any data previously collected. We invited participants in waves of 30–40 individuals to allow interviewing and analysis to occur together. The invitation included a participant information sheet and reply slip. We offered a £20 shopping voucher to thank them for participating and reimbursement of travel costs.

Participants were young adults aged 26–28 years. There were more male participants than females (15 versus 10). The majority (n = 18) reported having completed an undergraduate degree. Participants varied in their adolescent health risk behaviour engagement (low: n = 7, medium: n = 9, high: n = 9).^1^

To describe participants’ objective SEP background we used maternal educational status as this is a commonly used measure of socioeconomic background (Karvonen & Rahkonen, 2011).^2^ Using this system, participants were in one of two groups: 14 participants were in the low SEP background group (mothers without university degree) and 11 were in the high SEP background group (mothers with university degree). Critically, this SEP assessment represents one element of the complex make‐up of a person's socioeconomic background and this may not reflect how individuals understand their own SEP currently or at birth. We have used these labels as an indication of SEP background (not current SEP) to provide some context to the findings, aligned with our aim to explore the perceptions of the impact of parental SEP on young adults’ lives. Additionally, most of our participants saw university attendance as more common among their generation than their parents, suggesting that taking the participants’ own educational attainment level may have been less informative about their socioeconomic background. To explore current SEP, following Bolam et al. (2004), we asked participants about the meaning of SEP to them, generating rich data to allow us to explore the subjective meaning of socioeconomic circumstances and the relationship with health risk behaviours. These meanings were not described through ‘low/high SEP’ categories, but instead through participants’ experiences.

### Procedure

LT conducted in‐depth interviews with young adults between November 2018 and June 2019. Interviews took place in private meeting rooms (n = 19), over the phone (n = 5) or via Skype (n = 1). Interviews were recorded using an encrypted audio recorder.

We started the interviews by describing the terms socioeconomic position and socioeconomic status (SES) and asked participants how they understood their own, their parents’ and their peers’ SEP. Then participants were asked what they considered to be a ‘health behaviour’ and we discussed their own and their peers’ engagement with those behaviours during adolescence. Participants were then presented with a list of thirteen health risk behaviours to be discussed in the same manner. The range and types health risk behaviours were informed by previous work with two participant groups^3^ and were included in the ALSPAC questionnaires at age 15–16. The behaviours on the list included: physical inactivity; TV viewing; car passenger risk; cycle helmet risk; scooter risk; criminal/anti‐social behaviour; excessive alcohol consumption; tobacco smoking; cannabis use; illicit drug/solvent use; self‐harm; sex before age 16 and unprotected sex. We used a timeline as a visual aid to recall and reflection. We had in‐depth discussions on how background SEP related (or not) to health risk behaviours and their current socioeconomic circumstances. More detail on the procedures and interview guide is provided in Supplementary Material.

### Data analysis

Data analysis within grounded theory begins with ‘individual cases, incidents or experiences’ that progressively develop into abstract conceptual categories to ‘synthesise, explain and understand’ the data and to identify patterned relationships through ‘constant comparison’ (Charmaz, 1996). Ideas generated are verified by the data and categories are constantly ‘refitted’ to ongoing comparisons of incidents in old and new data (Charmaz, 1996). We went through ‘reduction’ whereby categories were transformed around central concepts until no further modification was needed (Charmaz, 1996). We employed the three stage coding procedure of *open coding*, *axial coding* and *selective coding* recommended by Strauss and Corbin (Strauss & Corbin, 1998). This technique allowed us to slowly build explanations while testing the ideas that emerged. As the lead researcher conducting the interviews, LT kept a research diary and analytic memos to practise reflexivity and reflect on all aspects of the design, data collection and analysis (Charmaz, 1996). LT coded the interview transcripts, with five of transcripts doubled coded by RC and DC. NVivo 11 software was used to organise the data.

### Ethics

Ethical approval was obtained from the ALSPAC Ethics and Law Committee and the Local Research Ethics Committees (REF: 66061). Participants provided written informed consent. Pseudonyms were used and all identifying information removed from transcripts.

## FINDINGS

**FIGURE 1:**
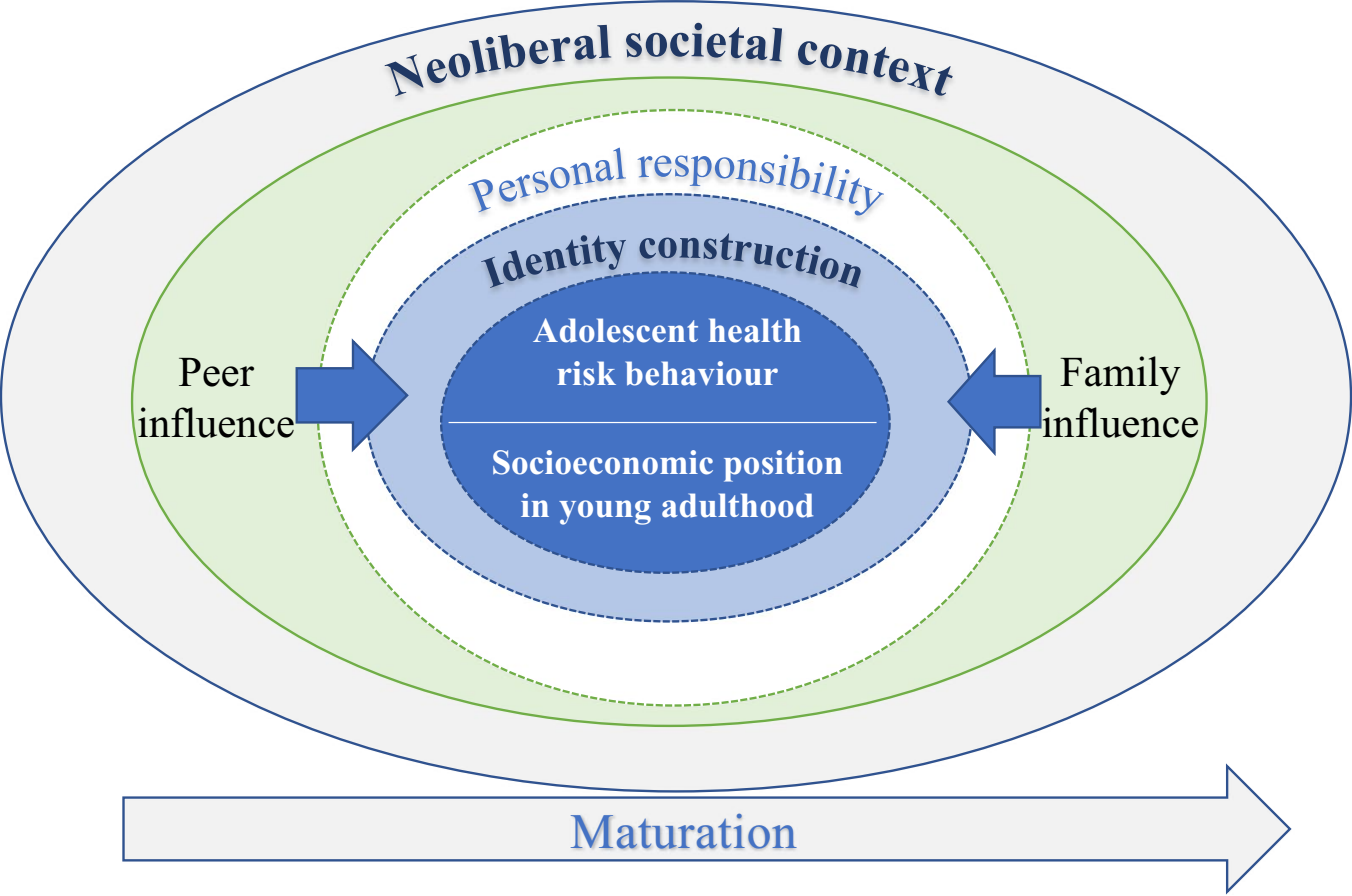
A grounded theory model illustrating young adults' perceptions of the relationship between adolescent health risk behaviours and socioeconomic position

We identified three interrelated categories that served as mechanisms for participants’ understanding of the relationship between adolescent health risk behaviour and SEP. The categories were *peer influence*, *family influence* and *personal responsibility*. Figure 1 illustrates these abstracted categories, the relationships between them and the neoliberal context within which they are situated in the form of a grounded theory model. Additional extracts of data which confirm the grounding of this model appear in Table S1 in Supplementary Material.

### Peer influence

Most participants referenced peer influence in relation to adolescent health risk behaviour. Some used the phrases ‘*peer pressure’* (Bradley, high SEP), ‘*acceptance*’ (Theo, low SEP) or ‘*wanting to look cool*’ (Christina, high SEP) to explain why they or their peers engaged in certain behaviours, with alcohol use, tobacco smoking, underage sex and illicit drug use being commonly cited. The most prominent conceptualisation of peer influence related to the idea that health risk behaviours were an almost inevitable symptom of being an ‘*immature*’ adolescent (Amber, low SEP) rather than something influencing or being influenced by SEP. This immaturity was discursively expressed in many ways, such as being ‘*naïve*’ (Connor, high SEP), feeling ‘*invincible*’ (Stacey, low SEP), ‘*getting carried away*’ (Stacey, low SEP), ‘*showing off*’ (Amber, low SEP), being ‘*insecure*’ (Christina, high SEP), being ‘*carefree*’ (Sasha, high SEP) or ‘*rebellious*’ (Nathaniel, high SEP) and doing ‘*classic teenage stuff*’ (Julian, high SEP). This perception of immaturity did not appear to depend on the participants’ SEP background. Peer influence was largely used in reference to substance use behaviours, not anti‐social behaviour or physical inactivity, for example. The arrow at the horizontal bottom of the model in Figure 1 depicts how engagement in health risk behaviours occurs within this wider context of maturation through adolescence.

Although peer influence was used to express a sense of equalisation during adolescence, how participants discursively represented peer influence provided some insight into how health risk behaviours and SEP interact for young adults. For instance, participants from relatively more advantaged upbringings often framed their own adolescent engagement in health risk behaviours in a positive light, as a ‘*learning experience*’ (Sasha, high SEP), ‘*having fun’* (Bradley, high SEP) or ‘*bonding*’ (Julian, high SEP). For them, adolescent health risk behaviours served an important function in the maturation process. These participants did not view their personal experiences as the result of *peer pressure*, but as peer *bonding*:

“When you drink you obviously lose your inhibitions… I think that was really important for a lot of my friendships… to have that experience and to… really bond over it I think… I don’t regret it at all.” (Emma, high SEP)

“I’d have between eight and fifteen drinks a night…with friends, going out, which I find good fun.” (Ali, high SEP)

When talking about *others’* engagement in the same behaviours, however, participants employed more judgemental explanations, such as feeling pressured to ‘*look cool*’ (Christina, high SEP). There was a contrast here between how these participants framed their own health risk behaviours (acceptable, chosen and potentially positive) and their peers’ health risk behaviours (showing weak character if the result of peer pressure, with the potential for negative effects). Distancing themselves from peers allowed presentation of self that circumvented the moral judgment surrounding health risk behaviours, allowing them to express neoliberal values of control and self‐responsibility. Participants from comparatively disadvantaged backgrounds who had engaged in health risk behaviours in adolescence tended to reflect on their personal experience with shame around the behaviour or succumbing to peer influence. Although these participants mirrored the immaturity narrative of their more affluent counterparts, there was little reference to peer bonding or positive functions of health risk behaviours among this group (Table S1, a):

“I personally was 15 when I lost my virginity, I don’t think we used protection… it’s just stupid.” (Stacey, low SEP)

“It’s just peer pressure, so you give in, cave in and do it [smoking cannabis].” (Gregg, low SEP)

Overall, peer influence was used to conceptualise a lack of a relationship between adolescent health risk behaviours and SEP. For some, this was expressed as the more positive peer *bonding*, with this perception more readily mobilised by more advantaged participants and usually as a framework for understanding their own experience as opposed to others. Conversely, *peer pressure* was used to understand others’ engagement in health risk behaviours (or personal experiences of less affluent participants), which was morally loaded. Both concepts broadly contributed to young adults’ understanding that health risk behaviours in adolescence were expected and normative due to immaturity, and thus not related to SEP. However, these divergent concepts begin to reveal class‐negotiated narratives surrounding participants’ perceptions of health risk behaviours, which as we will now explore, are closely tied to the family.

### Family influence

The family was an equally prominent lens for understanding the relationship between adolescent health risk behaviours and SEP. The family was more commonly adopted as a frame for understanding how health risk behaviours and SEP may be intimately connected during the transition to adulthood, in contrast to peer influence which was employed to show a lack of relationship. Through this lens, the idea of the ‘family’ becomes synonymous with socioeconomic background, or social class upbringing. For some, this related to class socialisation, with the understanding that socioeconomic circumstances of the family determined behaviour (Bourdieu, 1984; Singh‐Manoux & Marmot, 2005). For example, a common perception was that if an adolescent's parents smoke tobacco, they are themselves likely to do so, due to the normalisation of the behaviour. Those that spoke about this suggested that it was a pattern among lower socioeconomic groups:

“So if you’re in like… for example, have a single mum and live in a council estate and your mum, err, smokes a lot, then I guess there’s an element there at play, and possible that you would end up smoking a lot” (Julian, high SEP)

“I know it’s stereotypical, but the parents were the sort of people who were, living in a council house… probably didn’t have a job, the kids would come back and they’d be drinking six pints in front of the TV” (Damien, high SEP)

While Julian is theorising about how socialisation might work in relation to health risk behaviours such as smoking, his own and other participants’ experiences did not necessarily reflect this. For instance, Julian smokes cigarettes and he admitted that his parents were ‘*not very happy about it’* and were not tobacco smokers themselves, therefore, he is essentially resisting socialisation through his behaviour. Julian, who went to university and considers himself a young professional, associated certain health risk behaviours with working class families. This shows a structural understanding of links between health and SEP, however, it is done in a highly abstracted way that refers to others, not the individual experience (Bolam et al., 2004). Julian rationalises his own engagement in the same behaviours that he associates with working class groups, through an individualised lens of ‘*good experiences*’ and ‘*enjoyment*’. Stigma arises dependent on *who* is engaging in the behaviour, which is closely connected to the stigma of poverty. Julian engages in such behaviours outside of the stigmatised context of a low income ‘*council house*’ and his familial social class offers a level of protection from stigma that allows more legitimised engagement in health risk behaviours. Therefore, our argument is that several participants understood the family as key to the link between health risk behaviours and SEP and used it to explain classist frames of society. However, this perception was expressed in abstract terms and reserved for discussing others, whereas, one's own engagement in health risk behaviours was understood through individual agency.

Participants from less affluent backgrounds were acutely aware of the stigma related to their behaviours. Oliva saw her siblings as ‘*stereotypically working class’* as they engaged in cannabis use and one became pregnant as a teenager. Stacey recalls being surprised when ‘*wealthier’* friends engaged in ‘*naughty*’ behaviours, as she associated this with less educated, working class people like herself. Other participants expressed classist assumptions surrounding behaviours (Table S1, b):

“We did live in some ‘lower’ areas… and there is obviously a difference there with drugs being dealt and stuff.” (Amber, low SEP)

“If you’re from a family where there hasn’t been much money… you might think nothing of eating McDonalds three times a week. (Nigel, high SEP)”

These participants present an awareness of health risk behaviours as class markers. While some may accept these class identities, others, such as Olivia and Riley, described strategies for resisting class stigma through consciously avoiding engaging in health risk behaviours such as tobacco smoking, drug use, anti‐social behaviour and unprotected sex. It was the distinct absence of class stigmatisation that enabled their peers from ‘*richer*’ backgrounds to engage in adolescent health risk behaviours as a carefree exercise. Riley recalled peers from ‘*better backgrounds’* engaging in multiple health risk behaviours during his adolescence, which he attributed to access to resources to buy things like substances, but also the desire and ability to ‘*act older*’ without consequence:

“They almost didn’t really have to care because they knew that like their dad owned like a business or something and they would always get their money, or get a job that way.”

An additional way that family influence was conceptualised relates to our research question around how young adults saw their parents’ SEP as contributing to their own socioeconomic path. The family was described in terms of the material resources as well as social capital that may enable youth to transition into adulthood towards a particular SEP. A common example was that of university, with some noting the challenges of having parents who had not been to university themselves and so could not offer advice (Table S1, c). Many saw university attendance as more common among their generation, so there was the option to go onto higher education even if their parents had not. Participants from middle class backgrounds whose parent(s) had been to university saw higher education not as an opportunity, but as an expected path. Although cited as an advantage, this family influence was also expressed through control, expectation and pressure that came from parents. For these participants, going to university, even if they had no career in mind, was embedded within a value system of achievement and progression:

“It felt sort of what I was always destined to do… Well, not destined, but that was the path. Both my parents went to university. (Lucas, high SEP)”

“I don’t know what they would have said had I turned around and said I don’t want to go to university… I’d imagined they’d have said… this is a big decision for you not to go.” (Nigel, high SEP)

Nigel talked about the pressure he felt throughout school for everything to ‘*go to plan*’ and to have a good career. He acknowledged the financial, emotional and career support he had received from his family, but saw this as loaded with a certain amount of pressure to achieve. He attributed being ‘*addicted to work*’ as well as mental health issues partly to this pressure. Several other participants described a societal pressure to achieve, with many feeling a sense of failure for not being able to get a professional job and some experiencing low self‐esteem because of this. Therefore, while having familial socioeconomic advantage offers some protection during the transition into adulthood, whether that was material support or being free from stigma, this advantage at times reinforced the societal moral duty to be successful, which was not positive for some.

Participants from lower socioeconomic backgrounds also referenced how their family influenced their current SEP. However, these reflections related to family norms and behaviours as opposed to the parental support, resources and pressure that Nigel had experienced. Although Olivia had gone to university and had a reasonably well‐paid job, she was conflicted about how to describe her SEP, given her upbringing in a working class family:

“Researcher: Does that mean you would still sort of think of your socioeconomic position as the same as your parents?”

“Participant: I don’t think I would, but then, I think because I have come from that… Yeah, maybe I would still sort of relate that to, because that’s all I knew growing up, and it’s only like the past five years I’ve had a job and had my own money… for like twenty years of my life, it was always a bit of a struggle… I’m not out of that mind‐set yet.”

Olivia's conceptualisation of SEP as a ‘*mind*‐*set*’ reveals how family influence extends beyond material factors and remains embedded even in socially mobile young adults. She further acknowledged how her abstinence from health risk behaviours (except moderate engagement in alcohol) had, upon reflection, been motivated by her career goals as she claims, ‘*I didn't want to contradict myself*, *knowing I was going into healthcare*:

“Say if I was doing that [health risk behaviours] as a teenager… my parents would’ve probably tried to stop it… I guess looking back I would’ve thought if it was like the richer kids, their parents probably would have done something about it… have sorted it to then help them progress.’. Oliva wondered if she had engaged, would it have impacted on her career and educational progression”

This excerpt contributes strongly to our questions around the relationship between adolescent health risk behaviours, family SEP and young adult SEP. While being from a middle class family negates the stigma related to health risk behaviours, it also crucially offers protection against the damaging impact of such behaviours on the socioeconomic life trajectory. Middle class parents are perceptively more equipped to do ‘*something about it*’ if adolescents start to go off course as a result of their health risk behaviours. Riley, who had a similar upbringing to Olivia, extended this idea by claiming that he avoided certain health risk behaviours at school due to the possibility of getting ‘*side*‐*tracked*’, which was a greater risk for him and his working class peers than it was for ‘*richer*’ adolescents. A few other participants echoed this idea that middle class youth ‘*have more room to make mistakes*’ (Ali, high SEP) due to the ‘*safety net*’ (Nigel, high SEP) their families provided. This points to some understanding of the structural nature of inequalities and lack of opportunities and parental support for disadvantaged youth. However, this perception of family protection was bolstered by individual agency, as all participants positioned themselves as personally responsible for successfully transitioning into a healthy adult and not getting ‘*side*‐*tracked*’.

### Personal responsibility

The final lens through which young adults conceptualised adolescent health risk behaviours and SEP related to personal responsibility, individual agency and one's sense of self. Concepts such as ‘*drive*’ (Nigel, high SEP), ‘*determination*’ (Sarah, high SEP) and ‘*work ethic*’ (Joseph, low SEP) were adopted by several participants from varying backgrounds. Many participants strongly ascribed their own career and educational success to a level of personal determination and downplayed structural forces or family background. Similarly, those who were unemployed or in occupations they were not happy with expressed shame related to their personal failure. This contributed to some participants’ moral identity construction, connected to working hard and not relying on others:

“Honestly if you want something, the opportunity to get it is there. You just gotta be prepared to put the work in and the hours yourself.” (Joseph, low SEP)

“If you want something, you’ve got to work for it.” (Gregg, low SEP)

Other participants agreed that if you are determined and worked hard, you could achieve, regardless of your background. These perspectives highlight how the transition into adulthood is negotiated through embedded neoliberal ideologies. There were some participants who expressed examples of structural factors influencing their achieved SEP. For instance, several participants said they were ‘*lucky*’ (Christina, high SEP) or ‘*grateful*’ (Sarah, high SEP) when asked what they thought their SEP was. However, this was usually balanced with the view although your family background may put you at an advantage/disadvantage, there are ‘*enough opportunities out there*’ (Joseph, low SEP) that ‘*if you've got enough drive and determination*’ (Nigel, high SEP) you can achieve what you want to. This individualised concept is reflected in Figure 1, which conveys that while external influences are present in the outer circles, agentic expressions in the white inner circle remain central to young adults’ understanding of their achieved SEP.

This agentic discourse was also used to understand engagement in health risk behaviours and how these may negatively impact on adolescents’ life chances, revolving around the idea of ‘*personal responsibility*’ (Table S1, e):

“I would say personal responsibility is a big, big thing. If you’re going out, getting drunk three times a week… that’s not sort of, expressing responsibility.” (Theo, low SEP)

“If it’s something you want to try, try it…but I think you’ve got to know when you should start having responsibilities.” (Amber, low SEP)

Others echoed this perception by describing those who engaged in health risk behaviours such as drug use, vehicle risks and anti‐social behaviour as ‘*stupid*’ (Amber, low SEP) or ‘*cringey*’ (Sasha, high SEP). Young adults were expected to have ‘*grown out*’ (Bradley, high SEP) of these behaviours, particularly if they had a professional job or children. These extracts highlight that maturing is accompanied by increased personal responsibility to be an ‘ideal citizen’, which means not engaging in certain health risk behaviours as a morally negotiated practice. To reiterate our research question, our participants perceived health risk behaviours as potentially impacting on their socioeconomic life trajectory, namely if they continue into adulthood. As young people mature, they lose the legitimacy of immaturity and peer influence, as described previously, and so health risk behaviours become more intertwined with one's moral identity construction.

This moral imperative to be responsible was most pronounced for illicit drug use than other behaviours. Cocaine, MDMA and cannabis were frequently exemplified as central to moralistic understandings of health and the self. Several participants adopted phrases such as ‘*it just isn't me*’ (Rosie, low SEP), ‘*it never interested me*’ (Christina, high SEP) or ‘*it's not for me*’ (Joseph, low SEP). These phrases indicate that for some participants, engagement in certain health risk behaviours is strongly connected to their sense of ‘*me*’. Others reflected the idea of illicit drug use as an individual choice that was associated with an ‘*addict*’ identity (Emily, low SEP), which could easily ‘*get out of hand*’ (Ali, high SEP) and negatively impact on one's life trajectory. For some, the illegality of drug use informed this perception and allowed them to legitimise their engagement in hazardous alcohol use (Table S1, d). Young adults instead constructed alcohol behaviour through the idea of ‘*starting*’ to drink, with a common understanding about what is ‘*early*’ or ‘*late*’. Emma and Joseph had contrasting experiences, but both conveyed an expectation that they would start drinking during adolescence:

“Erm… and then when I got to uni, so I would have been nineteen, twenty, I was kind of bored, I was pretty done with it [alcohol] because I’d started so early…” (Emma, high SEP)

“But when you chat to other people now, they are like ‘wow that is really late’. A lot of my friends were like twelve, thirteen, fourteen, fifteen and I’m like ‘really?’” (Joseph, low SEP)

As alcohol use was described as more socially acceptable than health risk behaviours such as illegal drug use, it appeared it was not a significant marker of class identities or thought to negatively impact on socioeconomic opportunities. Instead, alcohol was seen as an expected normative behaviour for all adolescents during the transition to adulthood due to the perception that ‘*most adults drink in moderation’* (Theo, low SEP). These participants were ‘*getting started*’ (Andre, high SEP) with behaviour they would be expected to engage in as adults. In contrasting the embedded symbolic meanings within illicit drug use and alcohol use, we argue that young adults’ understandings of health risk behaviours in relation to SEP may differ dependent on the health risk behaviour. This frame of understanding could be applied to the other illegal/legal health risk behaviours, however, alcohol use and illicit drug use were the most readily adopted by our participants.

Although the family environment and material circumstances were used as explanations, personal responsibility was a prominent lens through which most young adults understood engagement in health risk behaviours during adolescence. Therefore, the potential negative effects of health risk behaviours on young adult SEP were commonly described through the pathway of neoliberal moral identity construction. This individualised discourse was pervasive among all participants regardless of background. Individualism was applied to the broad range of health risk behaviours, although much of what we have exemplified here relates to substance use behaviours, which participants continuously used to frame their understanding.

## DISCUSSION

The findings here represent the multiplicity of pathways through which young adults perceived adolescent health behaviour and SEP as connected. The family was an important factor that linked adolescent health risk behaviours and socioeconomic circumstances and therefore Bourdieu’s (1984) theories of socialisation and the habitus were useful in interpreting this category. Habitus is the generative schema whereby social structures become embodied within schemes of perception, providing the individual with a predisposed way of thinking, feeling and acting (Bourdieu, 1984). Socioeconomic circumstances determine habitus, which in turn determines behaviour or ‘practices’, with parents being the socialisation ‘agents’ of health risk behaviours (Singh‐Manoux & Marmot, 2005). Socialisation was a prominent lens through which our participants understood why working class youth are likely to engage in negative health risk behaviours. However, this was usually highly abstracted and used to talk about non‐specific others, not reflecting their own experience. This finding parallels other research in this area that found that to explain one's personal health within a class context would be unusual, as it would displace control and abdicate personal responsibility for health (Bolam et al., 2004).

Theoretical and empirical work has been done surrounding Bourdieu and the habitus related to how people ‘accept’ or ‘resist’ class identities they are born into (Thirlway, 2020). The stigma of poverty leads to a tension between wanting to reject respectability and to be respectable, and between aspiration and rejection of dominant values (Sayer, 2002), as exemplified in Olivia's mixed emotions surrounding her social mobility. Further, there was a recognition of ‘dual stigmatisation’ of poverty and health risk behaviour (Thompson et al., 2007), leading some to actively resist class stigma through avoiding health risk behaviours such as smoking tobacco, illicit drug use, unprotected sex and binge drinking, which they saw as ‘markers of broader difference in social background and cultural values’ (Graham, 2012). Avoiding these health risk behaviours further served as identity work, allowing them to present themselves as a ‘morally favourable self’ (Bolam et al., 2004) who had managed to overcome the negative behaviours expected of them.

Although some resisted personal class identification through their behaviour, they were also aware of tangible social and material resources through which class was ‘knowable’ to them (Bolam et al., 2004). These familial resources included knowledge of university and the ability to assist them if they were to experience negative consequences of health risk behaviours. These findings reflect Lawler’s (2005) description of the ‘narrative of lack’, in that some people are ‘deficient’ in both tangible material resources as well as ‘right ways of being and doing’ (Bourdieu, 1984), manifesting through the habitus, which is instilled at a young age. This narrative, Lawler posits, again reinforces notions of self‐responsibility and ‘robs subjects of narratives of moral worth’ (Lawler, 2005). The acknowledgement of ‘lack’ was reflected in our participants’ construction of their own individualised narratives of personal responsibility to not get ‘*side*‐*tracked*’, thus enhancing their sense of moral worth. Despite some of our participants consciously avoiding certain behaviours, which is undoubtedly a good thing for their health, celebrating these individualised actions of resistance runs the risk of undermining the power of material disadvantage and class stigma (Thirlway, 2020). Although participants highlighted their individual agency, socioeconomic circumstance of the family is clearly important in terms of life trajectories, health risk behaviour engagement and the interaction between them.

Peer influence was a pathway through which most participants legitimised health risk behaviour as a common feature of adolescence that was perceived as a part of growing up (Denscombe, 1993), and not something related to SEP. Through rationalising personal experiences as *‘classic teenage stuff’*, participants freed themselves from the ‘moral duty to be healthy’ (Blaxter, 1997), as their health risk behaviours were engaged in within the expected limits of normative adolescent behaviour. In this respect, peer influence was used to frame a sense of social equalisation in adolescence (West, 1997). In connection to this idea of normative youth behaviour, several participants showed how alcohol behaviour was inscribed within expectations of youth to a greater extent than other behaviours. Therefore, alcohol use was less illuminating of a relationship between health risk behaviours and SEP. These findings mirror sociological research that applied Bourdieu's concept of the habitus to alcohol and adolescence, which reported that UK drinking culture plays a major role in shaping alcohol behaviour, with there being a ‘shared habitus among young people that constructs heavy alcohol use as normative’ (MacArthur et al., 2016).

Finally, individualisation and neoliberal ideologies were interwoven through all participants’ discursive representation of health risk behaviours and SEP, which we have presented as a distinct category of ‘*personal responsibility’*. Many evoked this individualism through a discourse of choice, which subsequently diminished the notion of structural or environmental impacts on health (Midha & Sullivan, 1998). While participants could draw upon discursive resources to explain the structural relationship between health risk behaviours and SEP, individual choice was embraced as the overriding narrative. These representations reflected the ‘internalisation’ of neoliberal discourse that ‘delegitimises reliance on others’ and highlights the success of ideological strategies to divert from structural causes (Mackenzie et al., 2017). However, this internalisation, echoing the work of Pavis et al. (1998), does not suggest respondents are ‘dis‐embedded from social structures’, as these structures supplied the ‘meanings and choices’ and constrained their perceptions. Paradoxically, there was reference to how these ‘choices’ are not equally distributed (Wright & Laverty, 2010), balanced with individualised concepts of ‘*drive*’ and ‘*personal responsibility*’, allowing participants to experience a sense of control over their lives. Through these concepts, we found that what connected health risk behaviours and SEP for young adults was the idea of the successful and responsible adult, which in the West is positioned by neoliberal governmentality and was signalled by career status and control over health risk behaviours (Jeffrey, 2010).

There are some clear areas of future research. Firstly, our sample was recruited from a cohort study who are unusually comfortable with research. Secondly, owed to the fact that the ALSPAC birth cohort is overwhelmingly White British, our sample unfortunately reflected this. We were unable to sample based on ethnicity as we were already purposively sampling participants based on their level of health risk behaviour engagement, maternal socioeconomic position and gender. Recruiting more ethnically diverse samples would also allow exploration of intersectionality. We are committed to ensuring greater inclusivity in the production of data going forwards. Research in community settings (such as youth clubs) may aid this inclusivity and encourage greater exploration of inequalities and health risk behaviours in relation to place, which was an aspect unexplored in our project.

## CONCLUSIONS

Our study highlights young adults’ perceptions of the complex interconnectedness of health risk behaviours and socioeconomic life trajectories. Participants ratified classist frames of society though expressing health risk behaviours as class markers, often manifesting as class stigma, leading some participants to actively avoid certain adolescent health risk behaviours. Simultaneously, individualised, neoliberal ideologies in relation to both health risk behaviours and socioeconomic circumstances were interwoven through participants’ conceptualisations of their transition to adulthood. Given the problems with interventions that result from neoliberal ideologies, as explored in the introduction, programmes to improve adolescent health behaviour should instead focus on structural changes. This shift would avoid reinforcing ‘*personal responsibility*’ through behaviour change that serves to marginalise and maintain social inequalities.

## AUTHOR CONTRIBUTIONS

**Laura Tinner**: Conceptualization (equal); data curation (lead); formal analysis (lead); funding acquisition (equal); investigation (lead); methodology (lead); project administration (lead); writing‐original draft (lead); writing‐review & editing (equal). **Deborah Caldwell**: Conceptualization (equal); formal analysis (supporting); investigation (supporting); supervision (supporting); writing‐original draft (supporting); writing‐review & editing (equal). **Matthew Hickman**: Conceptualization (equal); formal analysis (supporting); funding acquisition (equal); investigation (supporting); supervision (supporting); writing‐original draft (supporting); writing‐review & editing (equal). **Rona Campbell**: Conceptualization (equal); formal analysis (supporting); funding acquisition (equal); investigation (supporting); methodology (supporting); supervision (supporting); validation (supporting); writing‐original draft (supporting); writing‐review & editing (equal).

## Supporting information

Table S1

Supplementary Material

## Data Availability

The data that support the findings of this study are available from the authors with the approval of ALSPAC. Restrictions apply to the availability of these data, which were used under licence for this study.

## ENDNOTES

^1^ Although we were able to sample according to different levels of health risk behaviour engagement we do not report the level of adolescent health behaviour engagement for each individual, as repeating the adolescent questionnaire in the interview would have been overly time consuming with the potential for recall bias.

^2^ To maintain confidentiality of the wider cohort study, we could not verify the self‐reported SEP background (participants’ reports of their mothers’ higher education attainment) using the data collected by ALSPAC.

^3^ Our choice of health risk behaviours was informed by discussions with two groups of adolescents through the DECIPHer (Centre for Development, Evaluation, Complexity and Implementation in Public Health Improvement) young people advisory group.
