# Supplementary material for: Understanding adolescent health risk behaviour and socioeconomic position: A grounded theory study of UK young adults
Source: Sociol Health Illn. 2021 Feb 26;43(2):528–44. doi: 10.1111/1467-9566.13240 (PMC8168338; doi:10.1111/1467-9566.13240)
Supplement: Supplementary file 1 — Table S1 [file SHIL-43-528-s002.docx]

# Supplementary material

# Table 1: Additional participant quotes with corresponding category and reference number

| Reference | Section/category | Participants and data |
| --- | --- | --- |
| a | Peer influence | It was good fun, pretty normal, what my friends were doing (Bradly, high SEP).  It started [heavy alcohol drinking] when I met him [boyfriend] really, when I was 16… it was really bad for my health and obviously like, reputations and stuff. It was just like, embarrassing, people knew you went to hospital and stuff (Sarah, low SEP). |
| b. | Family influence | I don’t know, maybe, like I know my friend, whose always been from a bit of a wealthy family, they’re not ridiculously rich but, like her mum and dad used to give them vitamins and things like that, and tablets, yeah vitamin tablets, so yeah, and she’s always got lovely hair and her skin’s always been lovely, so it might be that, and I guess having a healthy balanced diet, instead of I dunno like take-aways, like cheaper option of foods, I suppose it could be that as well (Stacey, low SEP).  I make everything from scratch, and I’m thinking, now that I look back, I’m thinking it’s because their parents were working long hours, erm, they didn’t have time to cook, so it was just the easiest thing, just because microwave mealing, erm, or get a takeaway. Just, what was easier (Sasha, high SEP). |
| c. | Family influence, parents had not been to university | My dad didn’t have a clue about, uni, like he wunt have been good with advice about uni, coz he’s quite a lot older, he was born in the forties, so back in those days I don’t think there was much of an option of going to uni, he’s from quite a lower-class family anyway. And then my mum, she’s not very emotional, so we don’t, we don’t have a bad relationship, but she wasn’t the type of mum I’d talk to about stuff like that anyway. I probably spoke to my teachers about it more. And they were quite supportive. Yeah (Olivia, low SES).  Erm… obviously they’ve never been so they dint really know, how, what to guide me on what they’d done or anything like that, because it’s all new to them as well, so (Rosie, low SES).  Perhaps because our families… our parents would have been people that did similar sort of paths, go to school, went onto university, it was kind of the done thing in our group (Christina, high SEP) |
| d. | Personal responsibility, illegality of certain health risk behaviours | I do know some people who do drugs, and I’m like well it’s illegal, you’re breaking the law, and that bothers me as well, the fact that it’s illegal. Like I know drinking’s not good for you, but it’s legal, so yeah, I find that it’s yeah, morally I can drink, but morally I can’t take drugs as well, coz it’s illegal, and they are bad for you, but yeah (Stacey, low SES).  Yeah I think part of the drug thing for me was, erm, the legality, I don’t know why I was, it sounds really boring and sensible, but I think, part of it was just I was just really kind of, clear that it was illegal and I didn’t want to do that, and I guess and then there’s on the other hand, alcohol, was legal-ish, but at that age (Christina, high SES).  It’s mainly the legal risk, I guess. It’s kind of not worth it in terms of… I have no burning desire to get involved in that. If anything were to go wrong, or get in trouble, I would feel quite stupid because it wasn’t really worth it (Clive, high SES). |
| e | Personal responsibility | I do consider myself to be in quite good health… like going to the gym regularly… and just being quite active generally, and just like making choices… not going for like take-aways and stuff, making food from home (Olivia, low SEP).  I’m very determined, and like you know, knowing what you can achieve is all in the mind-set…I probably used to [have harmful behaviors] but I’m trying to be more sensible, and especially with the career I want to go into (Sarah, low SEP).  I’m all for people trying things when you’re younger but if you go to the pub and there’s people in their 30s and 40s and their life is going to the pub and drinking and sniffing drugs… that’s their life… I think it’s a little bit sad (Sasha, high SEP).  I don’t want to be a bum… that’s the best way of putting it, I don’t want to be on benefits… I know quite a few people like that and I’m not being like that, I want to work and have a life… I chose to have a job and didn’t have kids, I didn’t drink I didn’t smoke (Emily, low SEP). |
