# Supplementary material for: Understanding adolescent health risk behaviour and socioeconomic position: A grounded theory study of UK young adults
Source: Sociol Health Illn. 2021 Feb 26;43(2):528–44. doi: 10.1111/1467-9566.13240 (PMC8168338; doi:10.1111/1467-9566.13240)
Supplement: Supplementary file 2 — Supplementary Material [file SHIL-43-528-s001.docx]

# Supplementary material for manuscript

## **Interview procedures**

All interviews were preceded with a friendly chat and a discussion of what to expect in the interview, with reassurance from the researcher that we were only interested in the participant’s experiences, that there were no right or wrong answers or judgement, and we could pause or stop the interview at any time for any reason. The researcher outlined each point on the consent form, which was signed before starting the interview. Interviews were recorded using an Olympus DS-3500 encrypted audio recorder.

A topic guide was developed aligned with the research question and study objectives. The interview guide went through a few iterations following consultation with the Original Cohort Advisory Panel (OCAP) of ALSPAC and the study team, who gave advice on wording and definitions. A pilot interview was conducted with a colleague who was approximately the same age as the study participants but was not in the recruitment pool.

The topic guide began with open questions about what the participant was currently doing in their life terms of employment, education or anything they deemed important. This was a rapport building exercise, but also served to set the tone for the interview as open and semi-structured. The first half of each interview consisted of discussing socioeconomic position as a concept, in particular: what they thought the concept meant, the socioeconomic position of their parents and peers as well as whether they had been aware of people having different socioeconomic backgrounds at school. Socioeconomic status was defined in the PIS and during the interview, although the other terms mentioned such as socioeconomic position, social position or social class were discussed with participants.

In the second half of the interview, participants were asked what they thought a health behaviour or health risk behaviour was and if they had any examples. The behaviours that participants highlighted were explored before going onto any health risk behaviours from an ALSPAC questionnaire they had been completed between the ages of 15 and 16 years. The health risk behaviours on the list included: physical inactivity; TV viewing; car passenger risk; cycle helmet risk; scooter risk; criminal/anti-social behaviour; alcohol consumption; tobacco smoking; cannabis use; illicit drug/solvent use; self-harm; sex before age 16; unprotected sex. We did not ask participants to repeat the health risk behaviours questionnaire they had completed at age 16. This decision was partly based on time restrictions of the interview, but also participants were told the interview would be a broad conversation of health risk behaviours based on their current reflections. Members of the OCAP highlighted that we should be cautious about asking participants about previous questionnaires as this might indicate that we were comparing what they said in interview to previous questionnaires or ‘checking’ their previous engagement in health risk behaviours. We kept this in mind and reassured participants we did not know their previous answers to questions and would not be checking them. For this reason, however, we were unable to provide each individual’s level of engagement in adolescent health risk behaviour in the results section next to the pseudonyms.

Participants were asked to reflect on their own and their peers’ engagement with health risk behaviour during adolescence. They shared their perspective on the extent to which this had impacted on their life now in relation to socioeconomic factors such as employment and education. They were asked when they started engaging in the health risk behaviours, if these behaviours had desisted and when, as well as how these behaviours related to other factors in their life at that time.

Participants were presented with a blank timeline of the past 13 years (2005 – 2018) with each point representing one year. This covered an age range of 13/14 years to 26/27 years. Having a visual aid was approved by the OCAP as it gave participants something to focus on during the interview and may help recall. We asked participants to plot significant moments in their life related to education, employment and risk behaviours, as well as anything they deemed important, such as having moved away from home or started hanging around a new group of friends. The researcher started the interview by asking what the participant was doing currently in 2018/19 and we worked backwards from there. Once the timeline had been populated, participants were asked about how the elements may be related as well as how this timeline may compare with their peers and parents. The participants were told that the timeline was meant to be a helpful aid and they did not have to use it. For this reason, participants used the timeline to varying degrees and telephone participants did not use it.

## **Topic guide**

**Health behaviours and young adulthood - Interview topic guide**

Version 0.7 24/12/2018

**Timeline**

- Tell me about your life at the moment.
- We are going to fill in a timeline with moments in your life you think are important (such as when you left school, if you got a job, went to college, **was it a different college/sixth form to your school/why,** changed jobs, went to university etc.) This information won’t be checked with anything you have previously told Children of the 90s and it is meant to be just a rough guide.
- How do you think these things compare to your parents/peers?
- Do you know how you would describe your socioeconomic status? (Ask if they know what this means and provide definition and examples if not) **Mention the idea of low, middle and high socioeconomic status and relationships to social class. Where would you put yourself?**
- How do you think that compares to your parents?
- Have you gone down a different education/employment route to them? Can you think of any reasons why?
- Is this something you ever thought about at school/when you were a teenager?
- Were you aware of any different groups at school based on socioeconomic status? In what ways?

**Multiple risk behaviour during adolescence**

- What do you consider to be health risk behaviours?
- Were your peers doing any of these behaviours when you were a teenager?
- Did you do any health risk behaviours when you were a teenager?
- Are you able to plot on the timeline when you started/stopped the behaviours?
- Have you continued any of these behaviours into your twenties?
- Which behaviours?
- To a lesser or greater extent?
- Can you think of any reasons why you have/haven’t continued these behaviours to the same extent into your twenties?
- Can you remember what other things were happening around these times? E.g. were you studying, did you have group of friends you did the behaviours with?
- Were any of your friends or people in your year doing any of these behaviours?
- Can you think of anything that might affect young people’s engagement in these behaviours? E.g. access to money to buy cigarettes?
- Here are some behaviours that we look at in the project. Do you recognise any of these as health behaviours?
- Can you think why you didn’t mention these to begin with?
- How do you think these behaviours compare in how you think of them?

**Reflections on the relationship**

- Do you think any of the things you have put on the timeline are related?
- Why do you think that is?
- Do you think that is different/the same for your peers? Why?
- Do you think your friends would have a similar timeline to you?
- Can you think of any others reasons why these risk behaviours during adolescence might affect your life in your twenties? (If participant thinks they do)
- Are there any other factors you think have contributed to what you are doing now?

**General prompts**

Risk behaviours included in Children of the 90s questionnaire as a prompt to ask participants if they recognise these as health risk behaviours.

- Physical inactivity
- TV viewing
- Car passenger risk
- Cycle helmet risk
- Scooter risk
- Criminal/anti-social behaviour
- Alcohol consumption
- Regular tobacco smoking
- Cannabis use
- Drug/solvent use
- Self-harm
- Sex before age 16
- Unprotected sex

**Generic probing questions will be ask in conjunction with the above, such as:**

- Can you tell me more about that?
- What exactly do you mean by that?
- Can you give me some examples?
- Is there anything more you want to say on that?
- Why do you think that is?
